# Restless Legs Syndrome/Willis–Ekbom Disease Is Prevalent in Working Nurses, but Seems Not to Be Associated with Shift Work Schedules

**DOI:** 10.3389/fneur.2018.00021

**Published:** 2018-01-29

**Authors:** Siri Waage, Ståle Pallesen, Bente Elisabeth Moen, Bjørn Bjorvatn

**Affiliations:** ^1^Department of Global Public Health and Primary Care, University of Bergen, Bergen, Norway; ^2^Norwegian Competence Center for Sleep Disorders, Haukeland University Hospital, Bergen, Norway; ^3^Department of Psychosocial Science, University of Bergen, Bergen, Norway; ^4^Centre for International Health, University of Bergen, Bergen, Norway

**Keywords:** RLS, WED, sleep, shift work disorder, shift work, night work

## Abstract

Insomnia and excessive sleepiness are among the most commonly reported sleep problems related to shift work. Sleep-related movement disorders have, however, received far less attention in relation to such work schedules. The objective of this study was to investigate the association between different shift work schedules and the prevalence of Restless legs syndrome/Willis–Ekbom disease (RLS/WED) in a large sample of Norwegian nurses. Our hypothesis was that shift working nurses would report higher prevalence of RLS/WED compared to day workers. A total of 1,788 nurses with different work schedules (day work, two-shift rotation, night work, three shift rotation) participated in a cohort study, started in 2008/2009. Four questions about RLS/WED based on the diagnostic criteria were included in wave 4 (2012). RLS/WED prevalence rates across different shift schedules were explored by the Pearson chi-square test. Logistic regression analysis was used to assess the association between RLS/WED and work schedules and shift work disorder (SWD) with adjustment for sex, age, marital status, smoking, and caffeine use. In total, 90.0% of the nurses were females, mean age 36.5 years (SD = 8.6, range 25–67). The overall prevalence of RLS/WED was 26.8%. We found no significant differences between the prevalence of RLS/WED across the different shift schedules, ranging from 23.3% (day work) to 29.4% (night work). There was a significant difference (*p* < 0.001) in the prevalence of RLS/WED between nurses having SWD (33.5%) compared to nurses not having SWD (23.8%). SWD remained significantly associated with RLS/WED in the adjusted logistic regression analysis (1.56, CI: 1.24–1.97). This study did not support the hypothesis. RLS/WED was associated with SWD, which might indicate that nurses vulnerable to shift work also are sensitive to other complaints related to a misalignment of the biological clock.

## Introduction

Shift work is associated with impaired health, whereof sleep problems are among the most commonly reported complaints (1, 2). Work schedules that involve night work are in particular associated with sleep problems, i.e., sleep onset and maintenance difficulties, reduced sleep duration, and excessive sleepiness during work (3). About 95% of shift workers working nights report sleep problems compared to 40% of day workers (4). Night workers are on duty during their biological resting phase and try to sleep during their biological active phase which has been proposed as a cause to sleep and health problems (5).

Even though insomnia and excessive sleepiness have been widely studied in relation to shift work, other sleep disorders, like for instance sleep-related movement disorders, have received little attention in this type of working population. One common sleep-related movement disorder is Restless legs syndrome/Willis–Ekbom disease (RLS/WED), which is characterized by unpleasant sensations in the legs represented by crawling, pulling and creeping feelings, and with an urge to move the legs (6). The diagnosis is also described as a circadian disorder of sensory-motor integration (7). The symptoms show a clear circadian rhythmicity and worsen during evening and night or while at rest. The symptoms typically occur at the transition between waking and sleep, disrupting sleep onset, or the return to sleep (8). The symptoms are relieved, at least for a short time, when moving, walking, rubbing, and massaging the legs (6). Prevalence rates of RLS/WED in Europe and the USA are reported to be between 2.4 and 10.8% (9). In the general population in Norway, a prevalence of 14.3% was reported, in which about half reported symptoms of moderate to very severe degree (10). Studies have shown that RLS/WED is more prevalent among females and increases with age (9).

Despite the fact that the occurrence of RLS/WED seems to be influenced by a circadian component, few studies have investigated the relationship between shift work and this disorder. The few existing studies on this topic show inconsistent results (11–13). Both sleep deprivation and fatigue might trigger RLS/WED symptoms; however, the impact of chronic circadian disruption on RLS/WED is unclear. Shift work is associated with reduced sleep duration (3), which may aggravate RLS/WED. Sleeping on a regular schedule, following proper sleep hygiene, eliminating caffeine before sleep, no smoking, exercising, and losing weight are some of the most common behavioral recommendations to ease and prevent symptoms of RLS/WED (7). Still, many of these recommendations are difficult to adhere to when working shifts. Notably, shift work is associated with higher BMI, sleeping at irregular times, use of caffeine to prevent sleepiness at work and the like (14). Against this backdrop, it is conceivable that shift work may be associated with increased rates of RLS/WED. Therefore, the present study aimed to investigate the association between different shift work schedules and the prevalence of RLS/WED in a population of Norwegian nurses. Our main hypothesis was that shift working nurses would report higher prevalence of RLS/WED compared to day workers.

## Materials and Methods

### Procedure and Participants

The data stemmed from the ongoing longitudinal cohort study “SUrvey of Shift work, Sleep and Health (SUSSH)” among Norwegian nurses. The first data collection was conducted during winter 2008/2009 (wave 1) when a sample of 5,400 nurses was randomly selected from the Norwegian Nurses Organisation’s (NNO) membership roll and invited to participate. The NNO includes most of the nurses in Norway. The initial sample comprised five equal strata based on the numbers of years since graduation from nursing school (0–11 months, 1–3 years, 3.1–6 years, 6.1–9 years, and 9.1–12 years). A total of 2,059 (response rate = 38.1%) completed the questionnaire at the first wave (2008/2009). In order to increase the study population, an additional sample of 906 newly educated nurses (response rate = 33.1%) was recruited in 2009. The total sample in wave 1, therefore, included 2,965 nurses. The nurses who responded to the first wave were invited to participate in annual follow-ups by receiving questionnaires sent by postal mail with prepaid envelopes for returning the completed forms. Up to two reminders were sent for each wave to those who did not respond. For each wave, the nurses received information about a lottery performed upon completing and returning the questionnaires, where 25 individuals would win a gift card with a value of 500 NOK (~70 US $). The present study reports findings based on data from the fourth (2012) wave. In 2012, 2,198 nurses completed the questionnaire, yielding a response rate of 74.1%. Nurses who reported that they were no longer working as nurses in wave 4 were excluded from the analyses, leaving a total study population of 1,788 nurses.

### Instruments

#### Demographics

The demographic variables were collected in wave 4, except for age and sex that were collected in wave 1. The questionnaire included data on the sociodemographic variables; marital status and caretaker responsibility for children in the household, work-related variables; work schedule (day only, evening only, two-shift including day and evening, night only, three-shift schedule including day, evening and night, and other schedules), type and percentage of full time position, and life-style variables; daily smoking (yes/no), daily number of cups of caffeine and body mass index. Due to low numbers, the nurses who were working “evening shift only” (*n* = 8) and “other schedules involving night shifts” (*n* = 57) were excluded from the analysis. Also nurses who did not report their work schedule (*n* = 19) were excluded.

#### Shift Work Disorder (SWD)

Shift work disorder was measured with three previous used questions (15, 16) based on the minimal criteria from the Diagnostic and Coding Manual from the second edition of the International Classification of Sleep Disorders (ICSD-2) (17). The questions were: (a) do you experience either difficulties sleeping or experience excessive sleepiness? (yes or no), (b) is the sleep or sleepiness problem related to a work schedule that makes you work when you normally would sleep? (yes or no), and (c) have you had this sleep or sleepiness problem related to the work schedule for at least 1 month? (yes or no). Participants were classified as having SWD when they responded “yes” to all three questions.

#### Restless Legs Syndrome/Willis–Ekbom Disease

RLS/WED was assessed with questions based on the ICSD-2 (17), and in line with the current third edition of this diagnostic system (8). Four questions were used to make a diagnosis of RLS/WED: (a) during the past 3 months, have you had an urge to move the legs or arms, usually accompanied with discomfort or unpleasant sensations in the legs or arms? (b) If yes, does this urge start or increase when you are resting, such as when lying or sitting? (c) Is the urge to move or the unpleasant sensation partially or completely relieved when you are moving, such as when walking or stretching? (d) Is the urge to move or unpleasant sensation worse late in the day or at night compared with the rest of the day? All four questions were rated “yes” or “no.” Only those who answered yes to all four diagnostic criteria were defined as suffering from RLS/WED. To assess the severity of symptoms, we also included a question about how much the nurses were bothered by the symptoms, rated on a five point scale ranging from “not bothered,” “mildly bothered,” “moderately bothered,” “quite bothered” to “very bothered,” and one question pertaining to how often they experienced the symptoms, rated on a five point scale ranging from “never,” “1 day or less per week,” “2–3 days per week,” “4–5 days per week,” and “6–7 days per week.” Severe RLS/WED was defined as answering “yes” on all four diagnostic criteria and being from moderately bothered to very bothered. Severe and frequent RLS/WED was defined as having severe RLS/WED and at least having these symptoms 2 days or more per week.

### Ethics

The study was approved by the Regional Committee for Medical and Health Research Ethics of Western Norway (REK-West, no 088.08). Informed consent in written form was obtained from all participants.

### Statistics

IBM SPSS Statistics 23 for Windows was used for the statistical analyses. The prevalence of RLS/WED in relation to the different types of shift schedules was explored by the Pearson chi-square test. In addition, logistic regression analyses were used to assess RLS/WED as the dependent variable with work schedule (day only, two-shift, night only and three-shift, day shift as reference) and SWD as predictors and with sex, age, marital status, smoking, daily cups of caffeine use as covariates.

## Results

Demographic characteristics are presented in Table 1. In total, 1,608 (90.0%) of the nurses were females and 179 (10.0%) were males. Mean age in 2012 was 36.5 years (SD = 8.6, range 25–67). Altogether, 12.5% were day only workers, 34.6% were two-shift workers, 8.1% were night only workers, and 44.8% were three-shift workers. Mean age of the day only workers was 39.1 years, 37.2 years among the two-shift workers, 36.0 years among the night only workers, and 35.3 years among three-shift workers, respectively. Close to three quarters (74.2%) of the nurses were married/cohabiting, and 54.5% had children living at home.

**Table 1:** Demographic characteristics of Norwegian nurses included in the study.

Sex (*n* = 1,787): female	90.0%
Age in years; mean and SD (*n* = 1,784)	36.5 (8.6)
Married/cohabiting (*n* = 1,762)	74.2%
Children living at home (*n* = 1,744): yes	54.5%
Smoking (*n* = 1,778): yes	8.9%
Cups of caffeine daily; mean and SD (*n* = 1,784)	3.5 (2.5)
Body mass index; mean and SD (*n* = 1,771)	25.1 (4.5)

The overall prevalence of RLS/WED was 26.8% (*n* = 470). In the total sample of nurses, the prevalence of severe RLS/WED was 12.4% and the prevalence of severe and frequent RLS/WED was 8.4%, respectively. There were no significant differences between the prevalence of RLS/WED and the different shift work schedules, ranging from 23.3% among day only workers to 29.4% among the night only workers (Table 2). Table 2 also presents the comparisons of severe RLS/WED and severe and frequent RLS/WED across the different work schedules, also showing non-significant associations. In terms of RLS/WED prevalence, there were no significant differences when comparing day only to the two-shift schedule (*p* = 0.17), day only compared to night only (*p* = 0.21), and day only compared to the three-shift schedule (*p* = 0.32).

**Table 2:** Prevalence of RLS/WED among nurses in different types of shift work schedules.

	Day only	Two-shift	Night only	Three-shift	*p*-Value
RLS/WED % (*n*)	23.3 (49)	28.2 (163)	29.4 (40)	26.8 (201)	0.51
Severe RLS/WED % (*n*)	11.7 (25)	14.2 (84)	11.6 (16)	11.9 (91)	0.57
Severe and frequent RLS/WED % (*n*)	9.4 (20)	9.8 (58)	5.1 (7)	8.0 (146)	0.28

*RLS/WED: experienced RLS during the last 3 months*.

*Severe RLS/WED: experienced RLS during the last 3 months and being at least moderately bothered*.

*Severe and frequent RLS/WED: experienced RLS during the last 3 months, being at least moderately bothered and with a frequency of 2 days or more per week*.

In the total sample of nurses, 30.7% (*n* = 532) were classified as having SWD. There was a significant difference (*p* < 0.01) in the prevalence of RLS/WED between nurses having SWD (33.5%) compared to nurses not having SWD (23.8%).

Table 3 reports the results from the logistic regression analyses with two predictors and five covariates where RLS/WED comprised the dependent variable. Only age and having SWD were positively associated with RLS/WED. Table 4 reports similar results from the adjusted logistic regression analyses with the same predictors and covariates where severe and frequent RLS/WED comprised the dependent variable.

**Table 3:** Logistic regression analyses with RLS/WED as the dependent variable among shift working nurses in Norway (*n* = 1,788).

Independent variables (*n* in crude analyses)	Crude^a^ OR (95% CI)	Adjusted^b^ OR (95% CI) *n* = 1,670–1,718	Adjusted^c^ OR (95% CI) *n* = 1,640–1,686
Sex			
Female (1,579)	1.00		
Male (176)	0.76 (0.52–1.10)		
Age (1,752)	**1.03** (1.01–1.04)		
Married/cohabiting			
No (451)	1.00		
Yes (1,301)	1.05 (0.82–1.34)		
Smoking			
No (1,590)	1.00		
Yes (156)	0.78 (0.55–1.12)		
Cups of caffeine daily (1,752)	1.02 (0.98–1.06)		
Shift work disorder			
No (1,192)	1.00		
Yes (531)	**1.61** (1.29–2.02)	**1.60** (1.28–2.01)	**1.56** (1.24–1.97)
Work schedule			
Day only (210)	1.00	1.00	1.00
Two-shift (577)	1.29 (0.90–1.87)	1.34 (0.92–1.94)	1.33 (0.91–1.93)
Night only (136)	1.37 (0.84–2.23)	1.46 (0.89–2.40)	1.42 (0.86–2.35)
Three-shift (751)	1.20 (0.84–1.72)	1.32 (0.92–1.90)	1.32 (0.91–1.90)

*^a^In the crude regression analyses, each independent variable was entered separately. Age and cups of caffeine were entered as continuous variables*.

*^b^Adjusted for sex and age*.

*^c^Adjusted for sex, age, marital status, smoking, and use of caffeine*.

*Significant values are shown in bold*.

*RLS/WED, restless legs syndrome/Willis–Ekbom disease*.

**Table 4:** Logistic regression analyses with severe and frequent RLS/WED as the dependent variable among shift working nurses in Norway (*n* = 1,788).

Independent variables (*n* in crude analyses)	Crude^a^ OR (95% CI)	Adjusted^b^ OR (95% CI) *n* = 1,700–1,728	Adjusted^c^ OR (95% CI) *n* = 1,650–1,696
Sex			
Female (1,579)	1.00		
Male (176)	0.77 (0.42–1.41)		
Age (1,752)	**1.04** (1.03–1.06)		
Married/cohabiting			
No (451)	1.00		
Yes (1,301)	1.11 (0.75–1.64)		
Smoking			
No (1,590)	1.00		
Yes (156)	**2.00** (1.24–3.23)		
Cups of caffeine daily (1,752)	**1.12** (1.06–1.19)		
Shift work disorder			
No (1,192)	1.00		
Yes (531)	**1.93** (1.37–2.72)	**1.94** (1.37–2.73)	**1.94** (1.37–2.74)
Work schedule			
Day only (210)	1.00	1.00	1.00
Two-shift (577)	1.06 (0.62–1.80)	1.11 (0.65–1.92)	1.06 (0.61–1.83)
Night only (136)	0.52 (0.21–1.25)	0.60 (0.24–1.46)	0.50 (0.20–1.23)
Three-shift (751)	0.84 (0.49–1.42)	0.98 (0.57–1.68)	0.92 (0.54–1.59)

*^a^In the crude regression analyses, each independent variable was entered separately. Age and cups of caffeine were entered as continuous variables*.

*^b^Adjusted for sex and age*.

*^c^Adjusted for sex, age, marital status, smoking, and use of caffeine*.

*Significant values are shown in bold*.

*RLS/WED, restless legs syndrome/Willis–Ekbom disease*.

## Discussion

The present study showed that nurses working in different shift work schedules reported similar prevalence of RLS/WED as compared to nurses working regular day work. This finding did not support our hypothesis that shift working nurses would report higher prevalence of RLS/WED compared to day workers. However, the overall RLS/WED prevalence of 26.8% was higher than expected when compared to the prevalence reported in the general population. In addition, nurses with SWD reported more RLS/WED compared to nurses without SWD.

Symptoms of RLS/WED worsen in the evening and during the night, and the circadian variation of symptoms constitutes a unique diagnostic feature (18). Rotating shift work and night work generally cause circadian rhythm misalignment and sleep problems including shortened sleep duration. Thus, such work hours were expected to accentuate sleep disturbances like RLS/WED. However, we found no differences in the prevalence of RLS/WED among the nurses across the different shift schedules. Very few studies have explored the relationship between shift schedules and symptoms of RLS/WED, and the few results reported to date have been inconsistent. The present results were at odds with a cross-sectional study among 780 automobile manufactory workers in Iran where a significant higher prevalence of RLS/WED in rotational shift workers compared to permanent day workers was reported. The highest prevalence rate in that study was found among rotating shift workers, mostly working night shifts. The authors conclude that rotational shift work may act as a risk or exacerbating factor for RLS/WED (13). The present findings are, however, in accordance with another Iranian study among textile factory workers where no significant difference in the prevalence of RLS/WED was found when comparing 210 shift workers to 204 non-shift workers (12). Similarly, one Greek epidemiological study also reported no association between RLS/WED and shift work (11).

The increase of RLS/WED in the evening and during the night is explained by several factors. First, the symptoms increase with the increase of sleepiness in the evening. A second contributing factor is the decrease in motor activity in the evening compared with the daytime (19). Nurses working in the evening and during the night are likely to report less sleepiness at these hours since they need to be physical active during their work hours due to work tasks and work demands. On the other hand, studies have demonstrated that the circadian oscillation in RLS/WED may occur independently of activity or sleep deprivation (18). Still, the urge to move the legs and any accompanying unpleasant sensation begin or worsen during periods of rest or inactivity such as lying down or sitting, and the symptoms are relieved by movement, such as walking or stretching, at least as long as the activity continues (9). Hence, it is possible that the workers in the present study suffering of RLS/WED would report less complaints when they work during the evening or night, since their work-related activity could reduce their symptoms. Thus, the specific occurrence of RLS/WED symptoms in shift workers should, therefore, be studied in more detail, for example, by sleep and RLS/WED diaries. Also, studies with longitudinal designs should be performed. In our study, the presence of a healthy worker effect is likely to be present and could hamper the ability to detect a relationship between RLS/WED and shift work. Furthermore, it should also be taken into account that the vast majority of the nurses in the present study were females working more than 50% of full position in addition to having child and family obligations. Hence, most of the sample represented a healthy working population of “double-working women.”

The prevalence of RLS/WED is reported to increase with increasing age, and the mean age of onset is during the third or fourth decade (20). Our findings were in line with this, as we found that RLS/WED was positively associated with age. In addition, we found an association between RLS/WED and SWD. SWD is a circadian rhythm sleep disorder that is due to a work schedule that overlaps with the individuals’ habitual sleep period. Main symptoms are insomnia during the sleep period and/or excessive sleepiness during the wake period (21). The prevalence of SWD has previously been reported to be high in Norwegian nurses (15). The fact that SWD was associated with RLS/WED in these nurses might indicate that nurses having one sleep disorder associated to shift work are more sensitive and are at greater risk to also experience other complaints related to their work schedule. Future studies should, therefore, investigate whether there might be some common vulnerability (e.g., genetic, health behavior, etc.) between these and other sleep-related disorders (22). It is also possible that people suffering of sleep-related problems like RLS/WED and SWD could be prone to associate their sleep problems to shift work.

Despite that our hypothesis about shift workers experiencing more RLS/WED compared to day workers was not supported, the overall prevalence of RLS/WED was high in the present population of nurses. Prevalence rates of RLS/WED throughout the Western part of the world vary between 2.4 and 15% (9, 23). In Norway, a mean overall prevalence rate of RLS/WED in the general population of 14.3% has been reported (10). RLS/WED is, however, more frequent in females compared to males (8), and females older than 35 years of age are twice as likely to suffer from RLS/WED compared to males (9). The mean age of the day workers was slightly higher than in the shift working groups. This may be one possible explanation why there was no difference in the prevalence of RLS/WED between day workers compared to the other shift working groups, as the prevalence of RLS/WED increase with age. It should, however, be noted that age was controlled for as a possible confounder in the analyses. Among females in two Scandinavian countries (Denmark and Norway together), a prevalence rate of 13.4% was reported (10). About 90% of the nurses in the present study were females, and in addition their mean age was 36.5 years, which can be one reason for the high prevalence. However, some of the variations in prevalence rates of RLS/WED across studies could partly be explained by methodological differences in the assessment of the disorder. Not surprisingly, when we looked at severe RLS/WED and at severe and frequent RLS/WED, the prevalence rates dropped. In fact, when assessing only severe and frequent RLS/WED, the prevalence rates were lowest in the night only group (although not significant), in contrast to the finding for the diagnosis of RLS/WED. This result is difficult to interpret and further studies on this topic are warranted. Still, the results might reflect the healthy shift worker effect (24) or some methodological artifacts, such a low number of observations within some cells.

### Limitations and Strengths

The present study was based on a large and homogeneous sample of nurses, which limits the influence from possible confounding variables. In addition, the study used a standardized and a well-validated instrument to diagnose RLS/WED. As a limitation, the response rate in the first wave was low, which may make the interpretation of the data and conclusions less generalizable. Still, it is an asset that the response rate in the wave where RLS/WED was assessed was as high as 74.1%. Another limitation of the present study was the fact that all data exclusively were based on self-reports, and not supplemented by, e.g., clinical interviews. Such interviews would strengthen the validation of the diagnosis and could also provide additional information about the onset of symptoms, family history of RLS/WED, possible use of medication, and so on. In addition, the symptoms that were included in the questions to assess RLS/WED are similar to symptoms of other medical and behavioral conditions such as myalgia, leg edema, leg cramps, and others, and could, therefore, be confused with RLS/WED (18). This could as such have contributed to an overestimation of the RLS/WED prevalence rate. However, since our aim was to study differences in prevalence across shift schedules, such an overestimation will likely not influence comparisons. Still, it should be noted that the RLS/WED diagnosis, according to the diagnostic criteria, is based on subjective assessment (8, 17). The overall prevalence of RLS/WED in the present study was high, but we found no difference between day workers and the shift working groups. A limitation of this study was that information about former shift work was not collected. Thus, it is likely to assume that the day worker group also consisted of some former shift workers. Furthermore, although the prevalence of RLS/WED was high, the number of persons with this diagnosis in some of the shift work groups was low, which may have reduced the statistical power of the study.

## Conclusion

The results of the present study did not support the hypothesis that shift workers report higher prevalence of RLS/WED compared to day workers. Still, the overall prevalence of RLS/WED in this population of Norwegian nurses was high. Furthermore, RLS/WED was associated with SWD, which might indicate that nurses vulnerable to shift work also are sensitive to other complaints related to a misalignment of the biological clock.

## Ethics Statement

The study was approved by the Regional Committee for Medical and Health Research Ethics of Western Norway (REK-West, no 088.08). Informed consent in written form was obtained from all participants.

## Author Contributions

SW: design of the study, collecting the data, data analysis, interpretation of the results, and drafting the paper. SP, BM, and BB: design of the study, interpretation of the results, and critical review of the paper. All authors have approved the final manuscript.

## Conflict of Interest Statement

The authors declare that the research was conducted in the absence of any commercial or financial relationships that could be construed as a potential conflict of interest.

## References

[1] Costa G. Shift work and occupational medicine: an overview. Occup Med (2003) 53:83–8. 10.1093/occmed/kqg045 12637591

[2] Harma M Kecklund G Shift work and health – how to proceed? Scand J Work Environ Health (2010) 36:81–4. 10.5271/sjweh.2902 20126970

[3] Akerstedt T. Shift work and disturbed sleep/wakefulness. Occup Med (2003) 53:89–94. 10.1093/occmed/kqg046 12637592

[4] Wedderburn AI Shiftwork and Health. Luxenbourg: Office for Official Publications of the European Communities (2000).

[5] Smith CS Robie C Folkard S Barton J Macdonald I Smith L A process model of shiftwork and health. J Occup Health Psychol (1999) 4:207–18. 10.1037/1076-8998.4.3.207 10431281

[6] Allen RP Picchietti D Hening WA Trenkwalder C Walters AS Montplaisi J Restless legs syndrome: diagnostic criteria, special considerations, and epidemiology. A report from the restless legs syndrome diagnosis and epidemiology workshop at the National Institutes of Health. Sleep Med (2003) 4:101–19. 10.1016/S1389-9457(03)00010-8 14592341

[7] Wijemanne S Jankovic J. Restless legs syndrome: clinical presentation diagnosis and treatment. Sleep Med (2015) 16:678–90. 10.1016/j.sleep.2015.03.002 25979181

[8] American Academy of Sleep Medicine. International Classification of Sleep Disorders. 3rd ed Darien IL, editor. Darian: American Academy of Sleep Medicine (2014).

[9] Garcia-Borreguero D Williams AM. An update on restless legs syndrome (Willis-Ekbom disease): clinical features, pathogenesis and treatment. Curr Opin Neurol (2014) 27:493–501. 10.1097/WCO.0000000000000117 24978636

[10] Bjorvatn B Leissner L Ulfberg J Gyring J Karlsborg M Regeur L Prevalence, severity and risk factors of restless legs syndrome in the general adult population in two Scandinavian countries. Sleep Med (2005) 6:307–12. 10.1016/j.sleep.2005.03.008 15923140

[11] Hadjigeorgiou GM Stefanidis I Dardiotis E Aggellakis K Sakkas GK Xiromerisiou G Low RLS prevalence and awareness in central Greece: an epidemiological survey. Eur J Neurol (2007) 14:1275–80. 10.1111/j.1468-1331.2007.01966.x 17956448

[12] Yazdi Z Sadeghniiat-Haghighi K Loukzadeh Z Elmizadeh K Abbasi M. Prevalence of sleep disorders and their impacts on occupational performance: a comparison between shift workers and nonshift workers. Sleep Disord (2014) 2014:870320. 10.1155/2014/870320 24977041 PMC4055012

[13] Sharifian A Firoozeh M Pouryaghoub G Shahryari M Rahimi M Hesamian M Restless legs syndrome in shift workers: a cross sectional study on male assembly workers. J Circadian Rhythms (2009) 7:12. 10.1186/1740-3391-7-12 19747404 PMC2749800

[14] Pallesen S Bjorvatn B Mageroy N Saksvik IB Waage S Moen BE. Measures to counteract the negative effects of night work. Scand J Work Environ Health (2010) 36:109–20. 10.5271/sjweh.2886 20011984

[15] Flo E Pallesen S Mageroy N Moen BE Gronli J Hilde Nordhus I Shift work disorder in nurses – assessment, prevalence and related health problems. PLoS One (2012) 7:e33981 10.1371/journal.pone.0033981 22485153 PMC3317447

[16] Waage S Moen BE Pallesen S Eriksen HR Ursin H Akerstedt T Shift work disorder among oil rig workers in the North Sea. Sleep (2009) 32:558–65. 10.1093/sleep/32.4.558 19413151 PMC2663659

[17] American Academy of Sleep Medicine. International Classification of Sleep Disorders, Diagnostic and Coding Manual. Westchester, Illinois: American Academy of Sleep Medicine (2005).

[18] Allen RP Picchietti DL Garcia-Borreguero D Ondo WG Walters AS Winkelman JW Restless legs syndrome/Willis-Ekbom disease diagnostic criteria: updated International Restless Legs Syndrome Study Group (IRLSSG) consensus criteria – history, rationale, description, and significance. Sleep Med (2014) 15:860–73. 10.1016/j.sleep.2014.03.025 25023924

[19] Michaud M Dumont M Selmaoui B Paquet J Fantini ML Montplaisir J. Circadian rhythm of restless legs syndrome: relationship with biological markers. Ann Neurol (2004) 55:372–80. 10.1002/ana.10843 14991815

[20] Picchietti DL Van Den Eeden SK Inoue Y Berger K. Achievements, challenges, and future perspectives of epidemiologic research in restless legs syndrome (RLS). Sleep Med (2017) 31:3–9. 10.1016/j.sleep.2016.06.007 27567163

[21] Zee PC Goldstein CA. Treatment of shift work disorder and jet lag. Curr Treat Options Neurol (2010) 12:396–411. 10.1007/s11940-010-0090-9 20842597

[22] Thun E Le Hellard S Osland T Bjorvatn B Moen B Mageroy N Circadian clock gene variants and insomnia, sleepiness, and shift work disorder. Sleep Biol Rhythms (2016) 14:55–62. 10.1007/s41105-015-0023-9

[23] Einollahi B Izadianmehr N. Restless leg syndrome: a neglected diagnosis. Nephrourol Mon (2014) 6:e22009. 10.5812/numonthly.22009 25695039 PMC4318015

[24] Knutsson A. Methodological aspects of shift-work research. Chronobiol Int (2004) 21:1037–47. 10.1081/CBI-200038525 15646248

